# Petrobactin Is Exported from *Bacillus anthracis* by the RND-Type Exporter ApeX

**DOI:** 10.1128/mBio.01238-17

**Published:** 2017-09-12

**Authors:** A. K. Hagan, A. Tripathi, D. Berger, D. H. Sherman, P. C. Hanna

**Affiliations:** a Department of Microbiology and Immunology, University of Michigan, Ann Arbor, Michigan, USA; b Life Sciences Institute, Department of Medicinal Chemistry, University of Michigan, Ann Arbor, Michigan, USA; c Life Sciences Institute, Department of Medicinal Chemistry, Department of Chemistry, Department of Microbiology and Immunology, University of Michigan, Ann Arbor, Michigan, USA; University of Wisconsin—Madison

**Keywords:** *Bacillus anthracis*, iron acquisition, siderophores, transporters

## Abstract

*Bacillus anthracis*—a Gram-positive, spore-forming bacterium—causes anthrax, a highly lethal disease with high bacteremia titers. Such rapid growth requires ample access to nutrients, including iron. However, access to this critical metal is heavily restricted in mammals, which requires *B. anthracis* to employ petrobactin, an iron-scavenging small molecule known as a siderophore. Petrobactin biosynthesis is mediated by *asb* gene products, and import of the iron-bound (holo)-siderophore into the bacterium has been well studied. In contrast, little is known about the mechanism of petrobactin export following its production in *B. anthracis* cells. Using a combination of bioinformatics data, gene deletions, and laser ablation electrospray ionization mass spectrometry (LAESI-MS), we identified a resistance-nodulation-cell division (RND)-type transporter, termed ApeX, as a putative petrobactin exporter. Deletion of *apeX* abrogated export of intact petrobactin, which accumulated inside the cell. However, growth of Δ*apeX* mutants in iron-depleted medium was not affected, and virulence in mice was not attenuated. Instead, petrobactin components were determined to be exported through a different protein, which enables iron transport sufficient for growth, albeit with a slightly lower affinity for iron. This is the first report to identify a functional siderophore exporter in *B. anthracis* and the *in vivo* functionality of siderophore components. Moreover, this is the first application of LAESI-MS to sample a virulence factor/metabolite directly from bacterial culture media and cell pellets of a human pathogen.

## IMPORTANCE

*Bacillus anthracis* requires iron for growth and employs the siderophore petrobactin to scavenge this trace metal during infections. While we understand much about petrobactin biosynthesis and ferric petrobactin import, how apo-petrobactin (iron free) is exported remains unknown. This study used a combination of bioinformatics, genetics, and mass spectrometry to identify the petrobactin exporter. After screening 17 mutants with mutations of candidate exporter genes, we identified the apo-petrobactin exporter (termed ApeX) as a member of the resistance-nodulation-cell division (RND) family of transporters. In the absence of ApeX, petrobactin accumulates inside the cell while continuing to export petrobactin components that are capable of transporting iron. Thus, the loss of ApeX does not affect the ability of *B. anthracis* to cause disease in mice. This has implications for treatment strategies designed to target and control pathogenicity of *B. anthracis* in humans.

## INTRODUCTION

Nearly all cellular life requires iron for growth. The redox potential of iron enables efficient electron transfer, making it a useful cofactor for proteins functioning in processes such as ATP generation, DNA replication, and the electron transfer chain. Harnessing iron for cellular growth, however, comes at a cost since free iron can undergo Fenton-type chemistry, whereby the reductive properties create damaging superoxide radicals (1). To prevent cellular damage, mammals tightly sequester iron with iron-binding proteins such as transferrin, ferritin, and lactoferrin. This reduces the pool of free ferric iron to less than 10^−18^ M and prevents iron from being readily accessed by invading bacterial pathogens (2). Iron acquisition is a growth-limiting step for nearly all bacterial pathogens and is frequently overcome by the production of siderophores (3). Siderophores are small molecules biosynthesized by bacteria during low-iron stress and are exported from the cell to scavenge ferric iron from the environment. In addition to siderophore biosynthesis operons, such pathogens must also encode dedicated proteins for unbound, or apo, siderophore export, iron-bound siderophore import, and iron release (3).

*Bacillus anthracis* is a Gram-positive, spore-forming bacillus with three operons encoding active iron acquisition systems: one for heme acquisition plus two siderophores, bacillibactin and petrobactin (4–6). Exposure to *B. anthracis* spores can result in inhalational, gastrointestinal, injectional, or subcutaneous anthrax, varying by the route of infection (7, 8). Using antigen-presenting cells as Trojan horses, *B. anthracis* can quickly reach the blood or lymph. Once in the blood, *B. anthracis* grows quickly, reaching septicemic loads as high as 10^8^ CFU/ml (7, 9, 10). Such rapid growth requires an ample supply of nutrients, including trace elements like iron.

Of the three systems encoded by *B. anthracis*, only the siderophore petrobactin has been shown to be required for virulence in murine models of inhalational anthrax (5). Since its description, the *B. anthracis* petrobactin biosynthesis operon, *asbABCDEF* (where “*asb*” indicates “anthrochelin biosynthesis” [5]), and petrobactin have been well studied (11) and explored as a drug target (12). Extracellular Fe^3+^-petrobactin is recognized by its surface receptor FpuA and imported by the action of redundant, ATP-binding cassette (ABC) transporters (for permeases, FpuB and FatC/D; for ATPases, FpuC, FpuD, and FatE) (13, 14).

In general, and relative to siderophore import systems, export systems of siderophores remain underdescribed. Currently, the mechanism of iron-free petrobactin export has not been identified. In described systems from other pathogens, siderophore export across the cell membrane can occur through dedicated proteins from transporter families, including the ABC, multifacilitator superfamily (MFS), and resistance-nodulation-cell division (RND) family (3).

ABC-type transporters consist of a permease and ATPase, which powers transport of the ligand via ATP. While import ABC genes typically encode separate polypeptides for each domain, exporter ABC transporters often have a single polypeptide containing both permease and ATPase domains (15). ABC-type siderophore exporters include the *Mycobacterium smegmatis* exochelin exporter ExiT, the *Salmonella enterica* salmochelin exporter IroC, and the *Pseudomonas aeruginosa* pyoverdine exporter PvdE (16–18).

MFS-type transporters are characterized by the MFS fold, 12 transmembrane helices split into the N and C domains. Transport is catalyzed by antiport or symport, whereby two or more ligands are transported in either opposing or parallel directions, frequently by the proton motive force (19). MFS siderophore exporters include *Staphylococcus aureus* NorA, *Azotobacter vinelandii* protochelin exporter CsbX, and the *Escherichia coli* enterobactin exporter EntS (20–22).

RND-type exporters resemble MFS transporters in structure but are often found in Gram-negative bacteria, where they form an export complex with a periplasmic protein and an outer membrane transporter (23). This is the case in *E. coli*, where following enterobactin export into the cytoplasm by EntS, extracellular export is facilitated by three RND-type efflux systems, each complexed with the outer membrane channel TolC (24). The cell membrane mycobactin and carboxymycobactin exporters in *Mycobacterium tuberculosis*, MmpL4/5, were the first reported RND-type siderophore transporters (25). While the outer membrane efflux channel is unknown, MmpL4 and MmpL5 require the action of cognate periplasmic accessory proteins, MmpS4/5, to facilitate biosynthesis and recycling of its siderophores (25, 26).

The *B. anthracis* genome does not contain evident siderophore accessory genes near the *asb* operon. (The receptor, permease, and ATPases are located elsewhere on the genome.) Thus, we developed a bioinformatics-based protocol to identify petrobactin exporter candidates. From this list, markerless deletion mutants were made and screened for their ability to grow in iron-limiting medium and to export petrobactin. To screen our mutants, we adapted laser ablation electrospray ionization mass spectrometry (LAESI-MS) for detection of bacterial metabolites, specifically petrobactin. Previous studies have used complex sample extraction techniques coupled with analytical methods, including thin-layer chromatography or mass spectroscopy, to screen for the presence or absence of petrobactin in a sample (4, 5, 13, 14, 27–29). However, such extensive sample preparation presented a bottleneck to high-throughput analysis and introduced experimental error, reducing our ability to quantify petrobactin and related molecules in a large sample set.

LAESI is an ambient ionization technique for mass spectrometry that uses an infrared laser and relies on the water present in the sample as a makeshift matrix to promote ion formation (30). The energy from the irradiated sample generates a fine plume of mostly neutral droplets. The analytes in the plume are subjected to charge transfer by a mist of electrosprayed buffer, which ionizes and propels them to the mass spectrometer for mass-to-charge (*m*/*z*) analysis. The majority of LAESI applications to date have focused on mass spectrometry imaging of plant and animal tissues, although recent studies have used LAESI to sample and identify bacterial metabolites from roadkill and soybean microbiomes (31–36). Thus, the application of LAESI-MS for the identification of the import/export gene partners for bacterial metabolites has not been explored.

Using LAESI-MS for high-throughput, high-accuracy analysis for petrobactin detection, we directly demonstrate petrobactin in the cells and supernatant of predicted exporter mutants. These results were confirmed using liquid chromatography–high-resolution electrospray ionization mass spectrometry (LC-HRESIMS). Through our novel application of LAESI-MS we identified the apo-petrobactin exporter (ApeX), a member of the RND-type transporter family, and observed the export of petrobactin components in its absence. We also show that these siderophore components can bind and transport sufficient iron for growth and are therefore relevant to disease in a murine model of inhalational anthrax.

## RESULTS

### Selection of candidate petrobactin exporters.

To select candidate petrobactin exporters, we took advantage of two types of bioinformatics data sets. First, most components of siderophore iron acquisition systems are expressed only during iron-starved growth. Multiple microarrays of *B. anthracis* Sterne growth under iron-limiting conditions exist, including growth in mammalian blood, within macrophages, and in iron-depleted medium (IDM) (37–39). Next, the *B. anthracis* genome was queried for homologues of known siderophore exporters (16, 17, 20, 21, 25) using their amino acid sequences in a position-specific iterative (PSI) BLAST search (40). The returned siderophore exporter homologues were then cross-referenced against iron-limiting growth microarray data, searching for those upregulated under one or more conditions. Through this protocol, we identified the candidate petrobactin exporters described in Table 1, including some transporters identified by their upregulation in iron-limiting growth, some siderophore exporter homologues with no regulation changes, and two genes (locus tag no. GBAA_1642 and GBAA_3296) encoding multidrug and toxic compound extrusion (MATE) family transporters (Table 1). The MATE transporters were hypothesized to be petrobactin exporters due to their proximity to the petrobactin operon in *Marinobacter* spp. (11). We focused on single gene targets and constructed unmarked in-frame deletion mutants in *B. anthracis* Sterne 34F2 for the candidate exporters in Table 2 using allelic exchange protocols established by Janes and Stibitz (41).

**TABLE 1:** Candidate petrobactin exporters

Family	Exporter homologue	Gene(s)^a^	PSI-BLAST (%)		Fold change						
			Coverage	Identity	IDM^b^			Blood, 2 h^c^	Mφ^d^		
					2 h	3 h	4 h		0 h	1–2 h	>3 h
MATE		*1642*			0.99	0.97	0.99	1.13			
		*3296*			1.01	1.10	1.02	0.90			

RND	*M. tuberculosis* Mmpl4/5	*1302*	69	25	0.97	0.89	0.94	1.15			
		*2407*	62	27	0.99	0.89	1.86	0.68			

MFS	*S. aureus* NorA	*0181^e^*	44	26	1.01	0.95	0.91	0.69			
		*0787^e^*	99	23	0.95	0.89	1.43	0.43			
		*2346*	80	22	1.07	1.08	1.06	0.94			
		*4961^e^*	37	33	0.96	0.75	0.91	1.02			
		*5668*	46	24	0.96	0.96	0.94	3.94		2.80	23.93
		*0835*	96	40	0.85	0.65	0.50	0.63			
		*1858^e^*	89	20	0.98	0.99	1.50	1.03		2.44	7.01
		*2004^e^*	89	24	0.99	0.94	0.84	0.76		−2.10	−2.10
		*3157*					1.58	4.12			

ABC	*E. coli* IroC	*0852^e^*	81	28	1.00	0.80	0.61	1.26		3.22	
		*0528^e^*	84	31	0.97	0.90	0.86	1.21			2.08
	*E. coli* EntS	*1652*	87	22	1.09	0.60	0.62	1.44			2.49
	*P. aeruginosa* PvdE	*5411^e^*	97	21	1.57	3.77	3.77	2.40			4.72
		*0349*–*0351*			1.84	5.06	2.83	5.63			3.22
		*3190–3191*			0.97	1.09	1.48	1.27	5.16	2.12	21.06
		*3531–3534*			2.32	6.92	6.09	6.43			2.24
		*4504*			1.10					3.83	
		*4595–4596*			3.01	28.07	19.38	11.82			6.60
		*5221–5222*			1.12					2.23	2.34

a GBAA_, *Bacillus anthracis* strain “Ames Ancestor” (taxid: 261594).

b Carlson et al. (38).

c Carlson et al. (37).

d Bergman et al. (39).

e Identified by multiple BLAST searches.

**TABLE 2:** Candidate petrobactin exporter phenotypes at 6 h postinoculation in IDM^a^

Family	Deletion strain type	Growth (OD_600_)		Catechols (% WT)^b^		Petrobactin (% WT)^b^	
		Avg	SD	Avg	SD	Avg	SD
MATE	Δ*1642*	0.52	0.05	85.58	9.29		
	Δ*3296*	0.51	0.02	99.30	11.23		
	Δ*1642* Δ*3296*	0.49	0.00	91.56	3.93	72.13	15.63
						
RND	Δ*1302*	0.53	0.05	94.56	13.61	99.67	37.31
	**Δ*2407***	**0.53**	**0.05**	**93.32**	**17.09**	**30.79**	**99.01**
	**Δ*****2407*****(pAH001)**^c^	**0.46**	**0.01**	**108.22**	**33.73**	**10.48**	**2.47**
	**Δ*****2407*** **p*****2407*****(pAH001)**^c^	**0.54**	**0.11**	**95.79**	**16.79**	**42.73**	**3.17**
	Δ*1302* Δ*2407*	0.51	0.01	90.43	8.68	0.00	0.00
						
MFS	Δ*0181*	0.51	0.04	94.08	9.33	23.00	4.04
	Δ*0787*	0.50	0.04	94.84	3.89	13.19	4.25
	Δ*4961*	0.52	0.04	114.93	27.71		
	Δ*5668*	0.62	0.04	113.87	15.68		
	Δ*0835*	0.64	0.02	100.47	11.71		
	Δ*1858*	0.63	0.02	94.74	13.37		
	Δ*2004*	0.45	0.10	102.33	34.11		
	Δ*3157*	0.51	0.01	89.59	5.98	13.56	65.05
	Δ*0835* Δ*1858*	0.48	0.06	96.25	12.00	22.38	14.72
	Δ*4961* Δ*5668*	0.48	0.01	96.68	5.59	30.44	3.49
						
ABC	Δ*0852*	0.64	0.03	95.85	21.66		
	Δ*0528*	0.52	0.07	94.66	8.55		
	Δ*1652*	0.52	0.03	109.82	14.57		
	Δ*5411*	0.61	0.06				
	Δ*4595–4596*	0.57	0.03	109.11	22.99		
	Δ*0528* Δ*0852* Δ*5411*	0.50	0.04	113.35	30.07	14.70	19.27

a Results for the Δ*2407* mutants are highlighted in boldface.

b Normalized to cell density (OD_600_) and presented as relative to the wild type (WT) set as 100%.

c Data compiled from two biological replicates.

### Screening candidate petrobactin exporters.

We reasoned that a *B. anthracis* Sterne mutant unable to export petrobactin would exhibit both reduced growth under iron-limiting conditions and reduced levels of petrobactin in culture medium. Accordingly, each of the mutants was screened for the ability to grow in IDM and for the presence of the petrobactin catechol component 3,4-dihydroxybenzoate (3,4-DHB). During growth of wild-type (WT) *B. anthracis*, 3,4-DHB can be measured in the culture medium IDM beginning at 3 h postinoculation (p.i.) using the Arnow’s assay, which detects catechols (42). When tested, all candidate export mutants both grew to wild-type levels in IDM and secreted wild-type levels of catechols (Table 2).

To explore a parallel redundancy in petrobactin export machinery as exists for petrobactin import (13), one triple deletion mutant and several double deletion mutants of the exporter candidates were generated—grouping them by transporter family. All double and triple deletion mutants similarly grew to wild-type levels in IDM and contained wild-type levels of catechol rings in the culture medium (Table 2). We surmised that the lack of a predicted phenotype could be due to either of two reasons: either our candidate identification strategy was flawed, or the detection of 3,4-DHB in the culture medium was not a valid proxy for export of intact petrobactin in the search for an exporter mutant.

### LAESI-MS detects intact petrobactin in culture media and cell pellets.

To address whether our mutants were exporting intact petrobactin and/or petrobactin components (e.g., 3,4-DHB and other biosynthetic precursors [4, 43]), culture medium from growth in IDM at 4 or 5 h postinoculation was queried using LAESI-MS. Previously, our laboratory has employed high-performance liquid chromatography (HPLC) for the detection of petrobactin in our sample supernatants (13, 14). This method is effective but not amenable to a high-throughput analysis of candidate exporter mutants. LAESI-MS provided the opportunity to conduct a sampling campaign of aqueous solutions (e.g., culture medium), while bypassing the sample preparation steps required by other MS techniques (44). During optimization of LAESI-MS for petrobactin analysis, we first determined the sensitivity of the instrument using a sample of pure petrobactin added to IDM at different concentrations (ranging from 0.004 to 1 mM). We observed a high mass accuracy of +0.2 ppm with a mass plus hydrogen ion ([M + H]^+^) peak at *m*/*z* 719.21. We further improved the LAESI peak shape to provide increased resolution and allowed more laser pulses to be averaged per MS scan by increasing the maximum injection time to 100 ms, which synchronized the LAESI pulse and the MS scan rate. By keeping the number of pulses per well at 20 with a frequency of 10 Hz, we obtained at least 4 data points per well. Each mutant is therefore represented by two time points from three independent growth curves, with technical replicates performed in triplicate.

**FIG 1:**
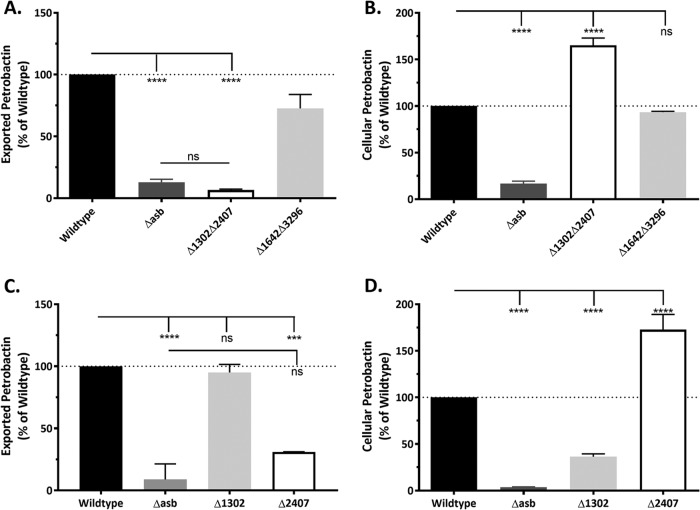
GBAA_2407 encodes a petrobactin exporter. LAESI-MS was used to detect petrobactin in spent media (A and C) and cell pellets (B and D) from cultures grown in IDM for 4 and 5 h. (A and B) MATE Δ*1642* Δ*3296* and RND Δ*1302* Δ*2407* double deletion mutants. (C and D) RND Δ*1302* and Δ*2407* single deletion mutants. The wild type and Δ*asb* mutant are present in all panels as positive and negative controls, respectively. Data were normalized to cell density at the time of sample collection and are presented relative to the wild type. Error bars represent standard deviations. Results are from two time points taken from three independent experiments measured in triplicate. Statistical significance was determined by two-way analysis of variance (ANOVA) with a Tukey’s multiple comparison posttest. ****, *P* ≤ 0.0001; ***, *P* = 0.0005; ns, not significant.

The processed data were normalized to the sample cell density (optical density at 600 nm [OD_600_]) and presented as a percentage of the wild type. We began by screening a selection of eight mutants, primarily consisting of double and triple deletions (Table 2). Petrobactin was absent from the culture medium of both the Δ*asb* negative-control mutant (no petrobactin biosynthesis) and the Δ*1302* Δ*2407* double mutant (RND-type transporters) (Fig. 1A). As an example of a candidate exporter mutant with wild-type-levels of petrobactin in the culture medium, and to disprove our hypothesis regarding their involvement in petrobactin export (11), we include the data from the Δ*1642* Δ*3296* mutant (MATE-type transporters) in Fig. 1.

We next hypothesized that an inability to export petrobactin would result in accumulation of the molecule within the bacterial cell. Cell pellets from cultures grown in IDM were obtained at 4 and 5 h postinoculation, washed twice with fresh IDM, resuspended in 30 µl water, and subjected to sampling by LAESI-MS. The data were normalized to cell density (OD_600_) and presented as percentage of wild-type petrobactin levels. As with the culture medium, Δ*1642* Δ*3296* cell pellet petrobactin levels were similar to wild-type levels, and the Δ*asb* mutant contained no petrobactin in the cell pellet. However, Δ*1302* Δ*2407* cell pellets accumulated petrobactin to levels greater than 400% of the wild-type level (Fig. 1B).

### GBAA_2407 **encodes a resistance-nodulation-cell division (RND)-type transporter that exports intact petrobactin.**

**FIG 2:**
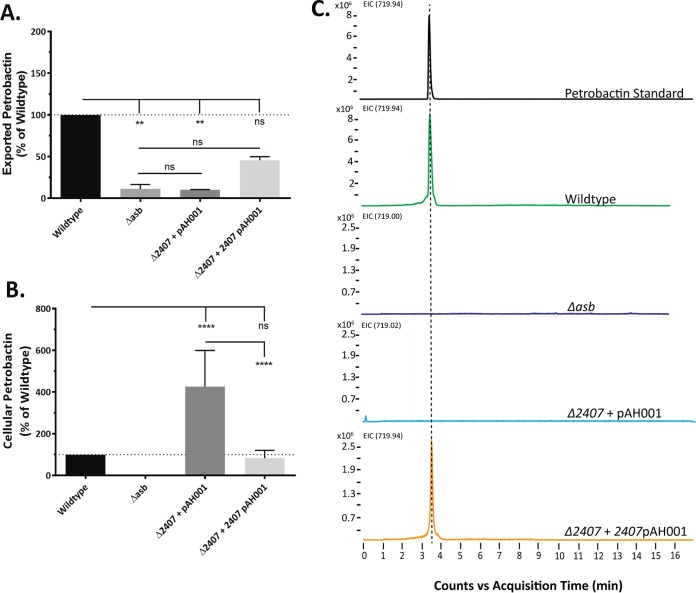
In *trans* complementation of the Δ*2407* mutant restores petrobactin export, reduces cellular accumulation, and is confirmed by HRESIMS. LAESI-MS was used to detect petrobactin in (A) spent media and (B) cell pellets from cultures grown in IDM for 4 and 5 h. Both panels A and B contain the Δ*2407*(pAH001) (empty vector) and Δ*2407* p*2407*(pAH001) (GBAA_2407 with native promoter) mutants plus the wild-type and Δ*asb* mutant positive and negative controls, respectively. Data were normalized to cell density at the time of sample collection and are presented relative to the wild type. The results reported are from two time points taken from three independent experiments measured in triplicate. Error bars indicate standard deviations, and statistical significance was determined by two-way ANOVA with a Tukey’s multiple comparison posttest. ****, *P* ≤ 0.0001; **, *P* < 0.005; ns, not significant. (C) Cultures were grown in IDM for 5 h, filter sterilized, frozen at −80°C, and lyophilized to dryness. Sample pellets were resuspended in methanol and subjected to HRESIMS. Shown are results from the petrobactin standard and wild-type, Δ*asb*, Δ*2407*(pAH001), and Δ*2407*(p2407) strains.

Since the initial LAESI-MS experiments were conducted on double deletion mutants, we returned to the single RND-type transporter deletion mutants (Δ*1302* and Δ*2407*) to determine which transporter(s) was responsible for petrobactin export. As with the double mutant, culture medium and cell pellets were analyzed by LAESI-MS for the presence of intact petrobactin molecules. Δ*1302* culture medium and cell pellets contained wild-type-like levels of petrobactin (Fig. 1C and D). However, the siderophore was not detected in Δ*2407* culture medium but was instead detected within the cell pellet, as we predicted for a mutant defective in petrobactin export (Fig. 1C and D). Conducting in *trans* complementation of GBAA_2407 under the native promoter (Δ*2407* p*2407*) rescued export of petrobactin into the culture medium (albeit not to wild-type-like levels [Fig. 2A]) and reduced its accumulation within the cell pellet (Fig. 2B). Analysis of the Δ*2407*(pAH001) vector control matched previous data (Fig. 2B). Collectively, these data strongly indicate that the GBAA_2407 gene product exports petrobactin.

As this appears to be the first application of LAESI-MS to measure siderophores directly in culture medium, we next sought to validate the presence or absence of petrobactin in culture medium from the wild type and Δ*asb*, Δ*2407*(pAH001), and Δ*2407* p*2407* mutants by LC-HRESIMS. Five-hour 25-ml IDM cultures were dried down, and the pellets were resuspended in methanol and analyzed. These data matched the LAESI-MS data, with petrobactin being detected in wild-type and Δ*2407* p*2407* culture media but absent from Δ*2407*(pAH001) and Δ*asb* media (Fig. 2C).

### The Δ*2407* mutant exports petrobactin components that maintain growth *in vitro*.

Despite the inability to export intact petrobactin (Fig. 1C), the Δ*2407* mutant maintained wild-type-like levels of growth in IDM as well as wild-type-like levels of the petrobactin component 3,4-DHB in the culture medium (Table 2). To explain the discrepancy between the absence of petrobactin (as measured by LAESI-MS) and the presence of 3,4-DHB in the culture medium, we hypothesized that when unable to export intact petrobactin, the Δ*2407* mutant degraded the molecule, exporting petrobactin components through an unidentified transporter.

First, we ruled out an alternate hypothesis that in addition to the export of petrobactin components, subdetectable amounts of petrobactin might be “leaking” from dead or dying Δ*2407* cells. Petrobactin accumulation within Δ*2407* cells could impact cell viability, resulting in elevated levels of cell death and leakage of petrobactin sufficient to rescue growth of the surviving cells. To test this alternate hypothesis, cultures grown in IDM for 4 h were stained for cell permeability using the LIVE/DEAD BacLight kit. Consistent with its poor growth in IDM, the Δ*asb* mutant had a low ratio of live to dead cells, while the wild type had a higher ratio, indicating less cell death (see Fig. S1A in the supplemental material). The Δ*2407* mutant maintained wild-type-like levels of cell viability, suggesting that there was not a gross increase in cell death and leakage in the absence of intact petrobactin export.

10.1128/mBio.01238-17.1 FIG S1  The Δ*2407* mutant resembles the wild type in cell viability and dose-dependent response to dipyridyl. (A) The Δ*2407*, wild-type, and Δ*asb* strains were grown in IDM for 4 h and stained for cell permeability. Data are presented as a ratio of live to dead cells and are the average and standard deviation from three independent experiments measured in triplicate. Statistical significance was determined by two-way ANOVA with a Tukey’s multiple comparison posttest. ****, *P* ≤ 0.0001; ns, not significant. (B) The Δ*2407*, wild-type, and Δ*asb* strains in IDM were supplemented with either 0, 50, 100, or 150 µM iron chelator 2,2-dipyridyl. Their growth was measured by OD_600_ every hour for 5 h. Data are representative of 3 independent experiments with triplicate measurements. Download FIG S1, TIF file, 0.3 MB. Copyright © 2017 Hagan et al. 2017 Hagan et al. This content is distributed under the terms of the Creative Commons Attribution 4.0 International license.

**FIG 3:**
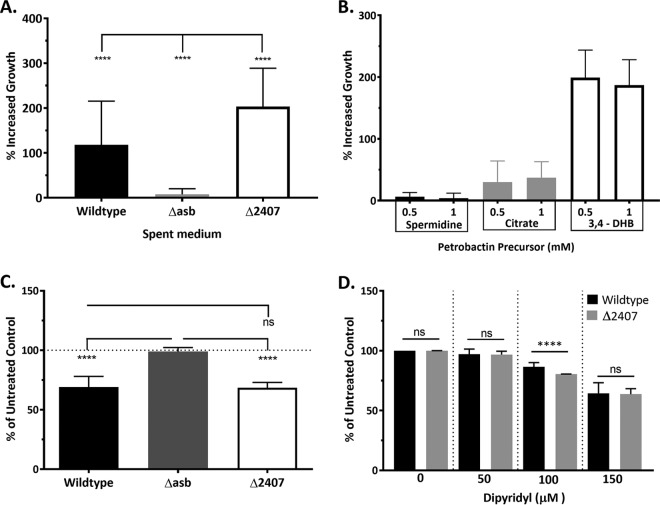
The Δ*2407* mutant exports petrobactin components that import iron but are less efficient than intact petrobactin. (A) Ten milliliters of spent medium from either the Δ*2407*, wild-type, or Δ*asb* strain was filter sterilized and added to 15 ml of fresh IDM inoculated with the Δ*asb* mutant. (B) Growth of the Δ*asb* mutant in 3 ml of fresh IDM was supplemented with either 0.5 or 1 mM concentrations of either citrate, spermidine, or 3,4-DHB. Data from panels A and B are presented as the percentage of increased growth (compared to the Δ*asb* mutant in IDM alone) at 5 h of growth and are the compiled average and standard deviation from three independent experiments. (C) The Δ*2407*, wild-type, and Δ*asb* strains were grown in IDM supplemented with 20 µM gallium sulfate at 0 and 2 h postinoculation. (D) The Δ*2407* mutant and wild type in IDM were supplemented with either 0, 50, 100, or 150 µM iron chelator 2,2-dipyridyl. Data from panels C and D are presented as the percentage of growth (versus the untreated control) at 4 or 5 h of growth, respectively, and are the compiled average and standard deviation from three independent experiments. Error bars indicate standard deviations, and statistical significance was determined by two-way ANOVA with a Tukey’s multiple comparison posttest. ****, *P* ≤ 0.0001; ns, not significant.

Next, we further hypothesized that petrobactin components can import sufficient iron to maintain Δ*2407* mutant growth in IDM. To test this, we both supplemented growth of the Δ*asb* petrobactin biosynthesis mutant in IDM with Δ*2407* spent medium and tested for the ability of the Δ*2407* mutant to import iron using gallium supplementation. Thus, the Δ*asb* mutant was supplemented with spent culture medium from the Δ*2407*, wild-type, or Δ*asb* strain (Fig. 3A). Both wild-type and Δ*2407* spent media increased Δ*asb* mutant growth: the wild type increased growth approximately 100%, and there was almost a 200% increase with Δ*2407* medium supplementation (Fig. 3A). The second approach employed the gallium toxicity assay. Gallium (Ga^3+^) resembles iron in both charge and size and can be imported by siderophores for incorporation as an enzymatic cofactor. However, while iron improves growth under iron-limiting conditions, Ga^3+^ inhibits bacterial growth since its redox potential is more limited than that of iron (45). Cultures grown in IDM were supplemented with 20 µM GaSO_4_ at 0 and 2 h postinoculation, and their growth at 4 h was compared to that of untreated controls. Growth of the Δ*asb* petrobactin biosynthesis mutant was not affected, since it lacks a siderophore, while growth of the wild type was inhibited about 30 to 40%. The growth of the Δ*2407* mutant in IDM was inhibited to similar levels to wild-type *B. anthracis*, indicating effective Ga^3+^ import and thus Fe^3+^ (Fig. 3C).

We reasoned that if the Δ*2407* petrobactin components had a lower affinity for iron, then growth in IDM by the Δ*2407* mutant would be limited by a lower concentration of the iron chelator 2,2-dipyridyl than wild-type *B. anthracis*. To determine if the Δ*2407* petrobactin components had the same or less affinity for iron as intact petrobactin, we cultured the Δ*2407*, wild-type, and Δ*asb* strains in IDM with either 0, 50, 100, or 150 µM dipyridyl. To quantify and compare across discrete growth curves, at 5 h postinoculation we calculated the percentage of growth of each sample compared to the untreated control. All strains demonstrated dose-dependent growth inhibition (Fig. S1B); however, at 100 µM, there was a statistically significant difference between the percentage of growth of the Δ*2407* mutant and that of the wild type (Fig. 3D). This confirms our hypothesis that the petrobactin components exported by the Δ*2407* mutant are not as efficient at iron scavenging as the intact molecule but still maintain some limited iron-binding abilities.

Petrobactin is composed of a central citrate moiety bearing two spermidine arms, each capped by a 3,4-DHB moiety (29). To identify which biosynthetic precursors of petrobactin maintain support of iron transport in *B. anthracis*, Δ*asb* mutant growth in IDM was supplemented with either 0.5 or 1 mM concentrations of citrate, spermidine, or 3,4-DHB. Spermidine did not improve Δ*asb* mutant growth in IDM, and citrate had marginal effects, averaging 30 and 37% increased growth at concentrations of 0.5 and 1 mM, respectively. 3,4-DHB, however, enhanced Δ*asb* mutant growth by nearly 200% at both concentrations tested (Fig. 3B). This piece of data does not identify the exact petrobactin components produced by the Δ*2407* mutant but does indicate that some (although not all) petrobactin-based components can assist in iron transport.

### Components could originate from truncated biosynthesis of petrobactin.

There are two, nonexclusive, hypotheses for the origin of extracellular petrobactin components (Table 3). First, petrobactin biosynthesis stalls, and the resulting precursors are exported. Second, petrobactin is synthesized and then degraded, possibly after sequestering cellular iron. To test the possibility that prebiosynthesized components are exported, mutants at various steps of petrobactin biosynthesis were tested for their ability to grow in IDM and for the presence of 3,4-DHB in the culture medium. All of the individual petrobactin biosynthetic gene disruption mutant strains tested (Δ*asbA*, Δ*asbB*, Δ*asbC*, Δ*asbD*, Δ*asbE*, and Δ*asbF*) had impaired growth in IDM (46): however, the impairment was not as severe as that of the Δ*asb* operon deletion mutant (Table 4; see Fig. S2A in the supplemental material). Additionally, all of the individual mutants except the Δ*asbF* mutant (AsbF is responsible for 3,4-DHB synthesis [47, 48]) had wild-type-like levels of catechols in the culture medium, suggesting export of biosynthetic precursors (Table 4; Fig. S2B). We note that AsbB is redundant for AsbA (43), although not vice versa. Thus, while the Δ*asbA* mutant still has a functional AsbB, enabling synthesis of intact petrobactin and increased growth, the Δ*asbB* mutation cannot serve a redundant function (43). See Table 3 for the predicted truncated products of each *asb* disruption mutant.

10.1128/mBio.01238-17.2 FIG S2  Petrobactin components from truncated biosynthesis are exported and supplement growth of the Δ*asb* mutant. (A) Individual Δ*asbA*, Δ*asbB*, Δ*asbC*, Δ*asbD*, Δ*asbE*, and Δ*asbF* petrobactin biosynthesis mutants, the wild type, and the Δ*asb* mutant were grown in IDM. (B) The strains were assayed for the export of catechols at 5 h. (C) Culture medium from each of the mutants in panel A was filter sterilized at 5 h postinoculation and used to supplement the growth of the Δ*asb* strain in IDM. (D) The difference in growth at 5 h was calculated as the percentage of increased growth relative to the Δ*asb* mutant in IDM alone. Growth curves (A and C) are representative of three independent experiments, and data (B and D) are compiled from three independent experiments. Error bars indicate standard deviations, and statistical significance was determined by two-way ANOVA with a Dunnett’s multiple comparison posttest. ****, *P* ≤ 0.0001; ns, not significant. Download FIG S2, TIF file, 0.7 MB. Copyright © 2017 Hagan et al. 2017 Hagan et al. This content is distributed under the terms of the Creative Commons Attribution 4.0 International license.

**TABLE 3:**
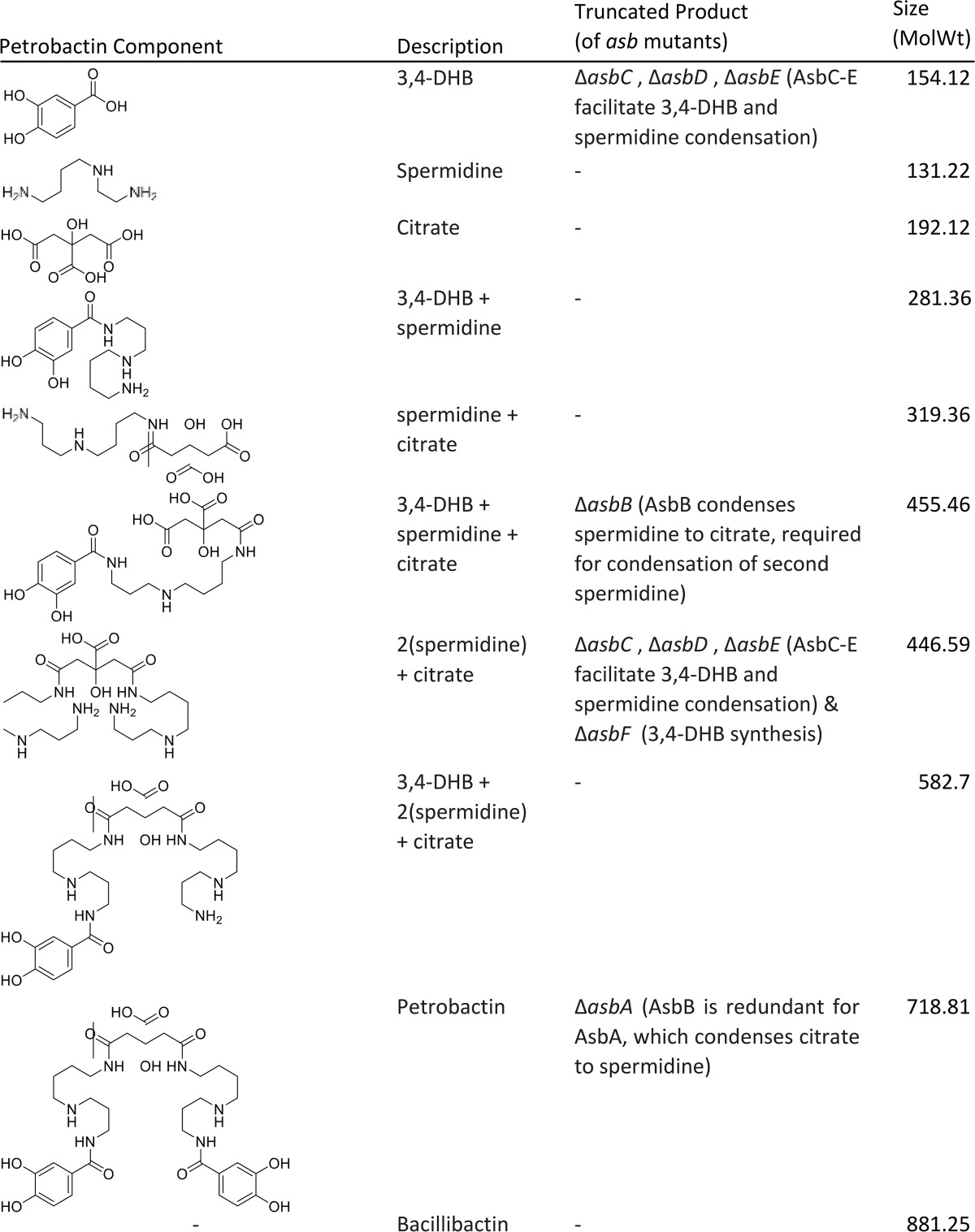
The structures and molecular weights of predicted petrobactin components present in individual *asbA* to -*F* knockout mutants^a^

a Nusca et al. (43).

**TABLE 4:** Growth of individual Δ*asbA* to *-F* disruption mutants in IDM

Strain	Growth (OD_600_)^a^		Catechols (% WT)^b^		% increase with Δ*asb* supplement^c^	
	Avg	SD	Avg	SD	Avg	SD
Wild type	0.55	0.03	100	0	53.14	16.73
Δ*asb* mutant	0.18	0.03	40.88	7.93	0.02	3.16
Δ*asbA* mutant	0.45	0.05	140.4	9.92	44.77	26.27
Δ*asbB* mutant	0.29	0.02	135.4	5.04	30.01	14.10
Δ*asbC* mutant	0.2	0.09	150.8	22	64.35	21.95
Δ*asbD* mutant	0.29	0.07	151.6	28.72	39.86	17.51
Δ*asbE* mutant	0.33	0.07	157.9	35.55	39.47	12.93
Δ*asbF* mutant	0.25	0.06	37.82	18.11	24.27	16.99

a In IDM at 5 h postinoculation (p.i.).

b Measurement of secreted petrobactin components at 5 h p.i.

c Δ*asb* mutant in IDM supplemented with the indicated medium at 5 h p.i. The percentage of  increased growth is relative to the Δ*asb* mutant in IDM at 5 h p.i.

To determine whether the growth lag was due to the absence of petrobactin biosynthesis or the presence of nonfunctional petrobactin biosynthetic precursors (e.g., citrate, spermidine, etc.), we filter sterilized culture medium from each of the mutants at 5 h postinoculation to supplement the growth of the Δ*asb* mutant in IDM. The *B. anthracis* Δasb strain was grown in 15 ml of fresh IDM supplemented with either 10 ml of spent medium from each *asbA*, -*B*, -*C*, -*D*, -*E*, or *-F* mutant, the Δ*asb* mutant, or the wild type (Fig. S2C). To quantify and compare across discrete growth curves, at 5 h postinoculation, we calculated the percentage of growth of each sample compared to the untreated control (Table 4; Fig. S2D). Supplementation of Δ*asb* mutant growth with spent culture medium increased growth over the Δ*asb* mutant in IDM alone, with exception of the Δ*asb* negative-control culture medium.

To characterize the exported petrobactin components, spent IDM culture media from the wild type or Δ*asb* and Δ*2407* mutants were filter sterilized at 5 h postinoculation and frozen at −80°C. The supernatants were lyophilized to dryness, resuspended in methanol, and assayed by LC-HRESIMS. Data obtained from LC-HRESIMS were queried for the presence of petrobactin and the projected petrobactin components listed in Table 3. However, peaks at the predicted component sizes were not petrobactin related, as they were also present in the Δ*asb* control (data not shown). The identities and sources of the petrobactin components exported by the Δ*2407* mutant remain to be confirmed.

### Petrobactin components use an alternate receptor and maintain *B. anthracis* Sterne virulence.

**FIG 4:**
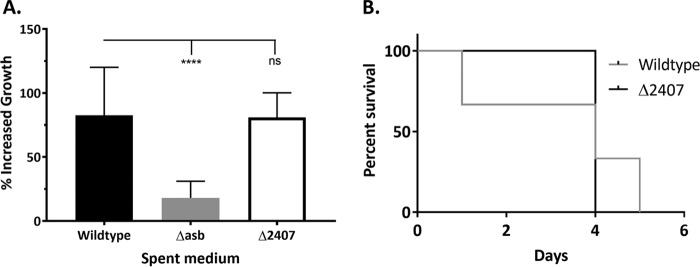
Petrobactin components use a different receptor from petrobactin and maintain *B. anthracis* Sterne virulence. (A) The Δ*fpuA* mutant was inoculated into 25 ml of IDM or 15 ml of fresh IDM with 10 ml of spent medium from either the Δ*2407*, wild-type, or Δ*asb* strain. Data are presented as the percentage of increased growth (compared to the Δ*fpuA* mutant in IDM alone) at 5 h of growth and are the compiled average and standard deviation from three independent experiments. Statistical significance was determined by two-way ANOVA with a Tukey’s multiple comparison posttest. ****, *P* ≤ 0.0001; ns, not significant. (B) Five 6- to 8-week-old, female DBA/J2 mice (Jackson Laboratories) were infected intratracheally with 1.5 × 10^5^ spores as previously described (49). Mice were monitored for morbidity for 14 days postinfection and euthanized when moribund. Data are presented as the percentage of survival.

To gain insight into how petrobactin components may be transported by *B. anthracis* Sterne, we took advantage of the receptor for iron-petrobactin, FpuA. The *B. anthracis* Δ*fpuA* mutant strain biosynthesizes and secretes petrobactin but grows poorly in IDM due to an inability to take up iron-bound petrobactin (14). The Δ*2407* mutant was grown in IDM for 5 h, and the culture medium was filter sterilized and then used to supplement the growth of the Δ*fpuA* mutant. The Δ*fpuA* strain was inoculated into 25 ml of IDM or 15 ml of fresh IDM with 10 ml of spent medium from either the Δ*2407*, wild-type, or Δ*asb* strain. As expected supplementation with Δ*asb* spent medium, the negative control, did not improve growth of the Δ*fpuA* strain. However, supplementation with either wild-type or Δ*2407* mutant medium improved growth by about 75% (Fig. 4A). These data suggest a different receptor for petrobactin components. To assess whether FpuA can recognize any petrobactin components in addition to petrobactin, Δ*fpuA* mutant growth in IDM was supplemented with either 0.5 or 1 mM concentrations of citrate, spermidine, or 3,4-DHB. Neither 3,4-DHB nor citrate enhanced Δ*fpuA* mutant growth, while spermidine inhibited growth (see Fig. S3 in the supplemental material). The receptors and cognate uptake systems for these petrobactin components remain unknown at this point; however, our data indicate that they will be distinct from the intact petrobactin system.

10.1128/mBio.01238-17.3 FIG S3  Δ*fpuA* mutant growth in IDM is not enhanced by petrobactin biosynthetic precursors. Δ*fpuA* mutant growth in IDM was supplemented with either 0.5 or 1 mM citrate, spermidine, or 3,4-DHB. The difference in growth at 5 h was calculated as the percentage of increased growth relative to the Δ*fpuA* mutant in IDM alone. Data are compiled from three independent experiments. Download FIG S3, TIF file, 0.2 MB. Copyright © 2017 Hagan et al. 2017 Hagan et al. This content is distributed under the terms of the Creative Commons Attribution 4.0 International license.

Finally, we investigated whether petrobactin components exported by the Δ*2407* mutant can maintain its virulence in a mouse model of inhalational anthrax. Five 6- to 8-week-old female mice were infected intratracheally with 1 × 10^5^ wild-type or Δ*2407* mutant spores (49). Mice from both groups became severely moribund, requiring euthanasia, within 3 to 5 days (Fig. 4B). That the Δ*2407* strain, which does not export intact petrobactin, was capable of producing wild-type-like levels of disease suggests that intact petrobactin is not absolutely required for iron gathering within the host.

## DISCUSSION

In this work, we developed a bioinformatics-based protocol to generate a list of petrobactin export candidates (Table 1), deleted them one by one from the *B. anthracis* genome, and screened them for their ability to export petrobactin and grow in IDM (Table 2). To facilitate the direct high-throughput analysis of petrobactin and its components generated by select *B. anthracis* mutants, we employed a new mass spectrometry technique, LAESI-MS. We have thus developed a novel methodology and data processing capability for the detection of siderophores and other metabolites from intact cells with minimal sample preparation. Our application of LAESI-MS effectively identified GBAA_2407 as a gene encoding an RND-type transporter responsible for exporting petrobactin (Fig. 1C). We also established that in the absence of product 2407, termed the apo-petrobactin exporter (ApeX), petrobactin accumulates in the cell, while corresponding components are exported through an unknown mechanism (Fig. 1D; Table 2). We also show that petrobactin components retain the ability to retrieve iron for growth and employ an unknown receptor since they rescue growth in the absence of the known petrobactin receptor (Fig. 3C and 4A). Finally, these components are sufficient to enable a mutant that does not export intact petrobactin to cause disease in a murine model of inhalational anthrax (Fig. 4B).

The *apeX* gene encodes a resistance-nodulation-cell division (RND)-like transporter, which has 12 transmembrane domains and is ubiquitous across cell types. RND-type transporters frequently function as trimers and interact with other proteins to form export complexes that transport a variety of ligands. Our initial list of candidate genes included those encoding two RND-like transporters, GBAA_2407 and GBAA_1302. Both were identified in a PSI-BLAST search of the *B. anthracis* genome with the *M. tuberculosis* mycobactin exporter Mmpl4. In *M. tuberculosis*, Mmpl4 coordinates with a periplasmic accessory protein, Mmps4, to export and recycle mycobactin in a process that also involves a last step in mycobactin biosynthesis (25, 26). ApeX is distantly related to Mmpl4, with 62% identity over 27% coverage. Mmpl4 and ApeX have two regions of homology with sporadic conserved residues (see the blue portions of Fig. S4A and B in the supplemental material), many of which lie in transmembrane helices 2 to 7. A PSI-BLAST search for ApeX-like proteins from the *Marinobacter* genus (data not shown), members of whom also produce petrobactin, also revealed sequence similarity to transmembrane helices 2 to 7 (dark pink in Fig. S4A and B). Overlays of Phyre2 protein predictions indicate some structural conservation between Mmpl4 (purple) and ApeX (gray) transmembrane domains but none in the extracellular (pore) domains (Fig. S4C) (50, 51). As *B. anthracis* is a Gram-positive bacterium, ApeX lacks a docking domain to interact with outer membrane export complexes (Fig. S4C).

10.1128/mBio.01238-17.4 FIG S4  Predicted structure of ApeX. (A and B) Phyre2 predicted structure of ApeX. Homology between Mmpl4 and ApeX is strongest in two areas: (i) transmembrane helices 2 through 7 (TM1 to -7), extracellular beta sheets 3 to 6, and extracellular helices 4 through 7 (pink and dark pink) and (ii) transmembrane helices 8 through 12, extracellular beta sheets 7 and 8, and extracellular helices 9 and 10 (green). Blue indicates conserved residues, and dark pink indicates regions homologous in a PSI-BLAST search of ApeX against the *Marinobacter* group, members of whom also produce petrobactin (data not shown). (C) Overlays of Phyre2 protein predictions indicate some structural conservation between Mmpl4 (purple) and ApeX (gray) transmembrane domains but none in the extracellular (pore) domains, and ApeX is not predicted to have a docking domain. Download FIG S4, TIF file, 1.5 MB. Copyright © 2017 Hagan et al. 2017 Hagan et al. This content is distributed under the terms of the Creative Commons Attribution 4.0 International license.

It remains unclear whether ApeX requires additional accessory proteins to facilitate petrobactin export. However, we did not identify a candidate within an operon with *apeX*, and neither did a PSI-BLAST search of Mmps4 against the *B. anthracis* genome reveal homologous proteins to Mmps4. While there is a small 60-residue hypothetical protein downstream of ApeX (GBAA_2408), it has an unknown function and is smaller than the 140-residue Mmps4. Given that petrobactin can be fully synthesized *in vitro* by supplying purified enzymes AsbA to -E with 3,4-DHB, citrate, and spermidine, we hypothesize that an accessory protein is unlikely. However, whether petrobactin biosynthesis occurs in the cell cytoplasm by transient interactions or if the biosynthetic enzymes form a discrete complex remains unknown. It is possible that an accessory protein facilitates biosynthesis by anchoring a complex to the cytoplasmic side of the cell membrane next to the exporter.

Regardless of whether petrobactin biosynthesis or export requires an accessory protein, the absence of the exporter ApeX results in the accumulation of intracellular petrobactin and export of components, which enables *B. anthracis* to retain the ability to retrieve sufficient iron for growth. This phenomenon has been described in *E. coli* siderophore systems (21), as has the ability for 2,3-DHB or citrate to supplement growth in low-iron media (52, 53). Our data demonstrate that *B. anthracis* can scavenge iron similarly, using either 3,4-DHB or citrate (Fig. 3B). The ability of culture medium from the Δ*asbF* mutant (which lacks 3,4-DHB but produces citrate-spermidine products) to rescue growth of the Δ*asb* nutant was unexpected (Table 4; Fig. S2D). This, along with data indicating petrobactin-dependent enhancement of the petrobactin receptor mutant (Fig. 4A), suggests that *B. anthracis* can import iron bound by distinct petrobactin components, including those with 3,4-DHB and/or citrate iron-binding moieties. The observation that *B. anthracis* can import Fe^3+^-citrate is worthy of further studies since rapid *B. anthracis* growth can occur in the host’s bloodstream, which might contain ferric citrate (54). A 2004 study observed 3,4-DHB in *B. anthracis* culture medium and hypothesized that it occurred from incomplete petrobactin synthesis (4). Our results (Table 4; Fig. S2B) confirm its presence and role, but we lack sufficient data to rule out enzymatic degradation of petrobactin. In fact, we describe our observation as “petrobactin components” to underscore our inability to distinguish between biosynthetic precursors and enzymatically derived fragments.

**FIG 5:**
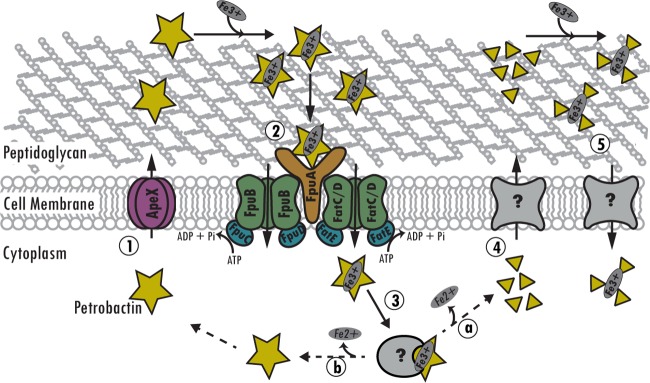
Model of petrobactin use in *B. anthracis*. (Step 1) Biosynthesized petrobactin is exported into the extracellular milieu through ApeX. (Step 2) Ferric petrobactin is recognized by FpuA to facilitate import through cognate permeases (FpuB or FatCD) and ATPases (FpuC, FpuD, and FatE). (Step 3) Iron is removed by either enzymatic cleavage (a) or iron reduction (b). (Step 4) Components are exported through an unknown transporter resulting from enzymatic cleavage and/or truncated biosynthesis. (Step 5) Petrobactin components bind iron and are reimported by an unknown mechanism. Dashed arrows indicate unknown mechanisms.

Our concept of petrobactin use by *B. anthracis* involves a model (Fig. 5) in which following biosynthesis of petrobactin, it is exported by the RND-type transporter ApeX (step 1). The ferric bound form is then recognized by FpuA for import by one of the ABC-type transporter permease-ATPase complexes FpuB/C, FpuB/D, or FatCD/E (step 2). Once in the cytoplasm, Fe^3+^ is released from holo-petrobactin by either enzymatic cleavage (step 3a) or by reduction (step 3b) (3), which would result in recycling of the siderophore and its components (Fig. 5). The citrate, spermidine, 3,4-DHB, and more complex petrobactin intermediates that originate from either truncated biosynthesis or cleavage of petrobactin are exported through an unknown mechanism (step 4), retrieve iron from the environment, and are reimported (step 5) (Fig. 5). If 3,4-DHB moieties bind the iron, then import occurs through the same import system, or if not, then it occurs through an unknown system. Iron is then released—probably by the reducing environment of the cytoplasm—and the components are probably continuously recycled. In the absence of ApeX, petrobactin accumulates in the cell, where it might be degraded after sequestering iron from cellular proteins, which then target it for degradation, and/or its biosynthesis is truncated, and the components are exported to retrieve iron. While *B. anthracis* encodes a second siderophore, bacillibactin, it is not observed in the supernatant until after 10 h of growth (55) (our experiments were performed between 4 and 6 h), and a corresponding peak was not observed in the LC-HRESIMS data (data not shown).

Siderophore degradation and export of the resultant fragments have been observed previously (21, 53, 56, 57), but whether those siderophore fragments are relevant to disease has remained unclear. Loss of enterobactin and/or salmochelin exporters in extraintestinal pathogenic *E. coli* (ExPEC) increased component export but reduced virulence (57). Virulence could be rescued by blocking enterobactin synthesis, which prevented the deleterious effects of biosynthesis and intracellular accumulation of two siderophores, while the third ExPEC siderophore, aerobactin, maintained virulence (57). *B. anthracis* virulence, however, relies on a single siderophore, petrobactin (5). Here, we demonstrate for the first time that siderophore components can gather sufficient iron in the host to cause disease. This observation raises questions to address in the future. For instance, it is unclear how the difference in affinity might affect iron transfer from iron-binding proteins. In addition, if siderophore components will suffice, what is the evolutionary value to biosynthesis of the entire molecule? Most importantly, in our minds, knowing if this phenomenon occurs with other pathogens is key to understanding what effect, if any, there is on treatment strategies designed to target siderophores.

If knocking out the exporter still results in export of siderophore components and no change in growth phenotype, it confounds research into identifying siderophore exporters, especially if the method used to measure the presence or absence of a siderophore does not have enough resolution to distinguish a component from the whole. This is probably the case in most research scenarios since hydroxamate and catechol colorimetric assays are the preferred methods for initial screening. This is what we encountered in our early work and why we initially turned from the low-resolution catechol assay to LAESI-MS—to measure petrobactin both in the culture medium and the cell. Adaptation of LAESI-MS for the detection and measurement of metabolites within the bacterial cell and in culture medium demonstrates it as a powerful tool. For instance, LAESI-MS could be applied to identify metabolic compounds undetected by other high-resolution detection techniques because they are either not amenable to the required extraction methods or involve low-throughput sample processing capabilities. LAESI-MS could also be adapted for the detection of bacterial or viral proteins or molecules directly from patient samples. This would enable a rapid diagnosis that bypasses costly sample preparations.

## MATERIALS AND METHODS

### Bacterial growth conditions and sporulation.

The strains used are described in Table S1 in the supplemental material. All mutants were derived from *Bacillus anthracis* Sterne 34F2(pXO1^+^, pXO2^−^) and generated by allelic exchange, as described by Janes and Stibitz (41). Complementation of GBAA_2407 was generated by PCR amplification and Gibson cloning (New England Biolabs) of the gene into pMJ01 under control of the native promoter (58). All necessary primers are listed in Table S2 in the supplemental material. Modified G medium was used for the generation of *B. anthracis* spores at 37°C for 72 h (59). Spores were collected at 2,800 rpm and then washed and stored in sterile water at room temperature following heat activation at 65°C. Bacterial strains were plated on brain heart infusion (BHI) (Difco) and grown in BHI at 30°C overnight. Overnight cultures were back-diluted 1:50 into fresh BHI and incubated at 37°C for 1 h. To remove contaminating iron and prepare the cells for growth in iron-depleted medium (IDM), the cells were pelleted at 2,800 rpm for 10 min (centrifuge 5810R; Eppendorf) and washed in 1 ml of fresh IDM five times (5). Twenty-five milliliters of IDM was inoculated at a starting optical density at 600 nm (OD_600_) of 0.05 and grown at 37°C with hourly measurements for 5 to 6 h. Strains containing the complementation plasmid were grown in the presence of 10 μg/ml chloramphenicol. Media and chemicals were purchased from Fisher Scientific or Sigma-Aldrich.

10.1128/mBio.01238-17.5 TABLE S1  Strains of *B. anthracis* Sterne 34F2 used in this work. Download TABLE S1, DOCX file, 0.01 MB. Copyright © 2017 Hagan et al. 2017 Hagan et al. This content is distributed under the terms of the Creative Commons Attribution 4.0 International license.

10.1128/mBio.01238-17.6 TABLE S2  Primers used to generate mutant strains used in this work. Download TABLE S2, DOCX file, 0.01 MB. Copyright © 2017 Hagan et al. 2017 Hagan et al. This content is distributed under the terms of the Creative Commons Attribution 4.0 International license.

### Catechol measurement.

The Arnow’s catechol assay was used to measure catechol rings in IDM (42). Beginning at 3 h postinoculation, 300 μl of each culture was pelleted at 13,000 rpm (centrifuge 5415R; Eppendorf) for 1 min and pipetted into three 40-μl aliquots in a clear 96-well plate. To each well, 40 μl of 0.5 M HCl, 40 μl of nitrate-molybdate reagent (10% sodium nitrate and 10% sodium molybdate), and 40 μl 1 N NaOH were added sequentially. The absorbance was measured at 515 nm in a SpectraMax M2 spectrophotometer and subtracted from a medium blank. Data are presented as a percentage of the wild type after being normalized according to cell density as measured by OD_600_.

### Cell viability.

Cell viability was measured using the LIVE/DEAD BacLight kit (Invitrogen). Briefly, 300 µl aliquots were taken from *B. anthracis* Sterne cultures grown in IDM for 4 h. The cells were pelleted for 1 min at 13,000 rpm, washed once, and resuspended in an equal volume of sterile phosphate-buffered saline. One-hundred-microliter aliquots were distributed into three wells of a 96-well black, clear-bottom microtiter plate. An equal volume of 2× Cyto9-propidium iodide was added to each well, and then the mixture was incubated in the dark for 15 min. Florescence was measured at 530 nm and 630 nm (excitation, 485) in a SpectraMax M2 spectrophotometer. Data are presented as a LIVE/DEAD ratio of 530/630.

### Gallium supplementation.

In IDM, each strain was used to inoculate two flasks. One was supplemented with 20 µM GaSO_4_ in 0.1% nitric acid at both 0 and 2 h postincubation, and the second was left untreated. They were grown at 37°C for 4 h. Data are presented as percentage of growth of the untreated control at 4 h: (OD_600_ of treated at 4 h/OD_600_ of untreated at 4 h) × 100.

### Culture medium supplementation.

For each strain, the appropriate number of flasks containing 15 ml IDM plus 10 ml filter-sterilized spent IDM were inoculated at identical starting optical densities at no less than an OD_600_ of 0.01. Spent culture media were obtained from IDM cultures grown as indicated above but which had their culture media collected at 5 h, filter sterilized with a 0.22-µm-pore filter, and stored at −80°C. The culture medium-supplemented cultures were grown at 37°C for 5 h. Data are presented as the percentage of increased growth relative to the fresh IDM-only control at 5 h: [(OD_600_ of treated at 5 h − OD_600_ of untreated at 5 h)/OD_600_ of untreated at 5 h] × 100.

### Dipyridyl and precursor supplementation.

Three milliliters of IDM was inoculated with the appropriate strain for a starting OD_600_ of 0.05 (WPA biowave CO9000 cell density meter) and supplemented with the indicated concentrations of either 2,2′-dipyridyl, citrate, spermidine, or 3,4-dihydroxybenzoate. Stock solutions were suspended in water and filter sterilized. Cultures were grown at 37°C with shaking for 5 h. Data are presented as the percentage of increased growth relative to the IDM-only control at 5 h: [(OD_600_ of treated at 5 h − OD_600_ of untreated at 5 h)/OD_600_ of untreated at 5 h] × 100.

### LAESI-MS.

For analysis, culture media and pellets were collected from cultures grown in IDM for 4 and 5 h. Culture media were separated from the cells by centrifugation and then frozen at −80°C until analysis. Pellets were washed in an equal volume of fresh IDM and frozen at −80°C. After thawing at room temperature, the pellets were resuspended in 30 µl of water for analysis. Twenty microliters of each sample was plated in triplicate wells of shallow 96-well plates and subjected to laser-based ablation. Here, the sample is heated by irradiation from a 2,940-nm infrared laser, leading to vaporization of molecules from the surface. The process initiates a dynamic equilibrium between energy deposition and consumption of the vaporization process. Any shift toward larger deposition of energy (such as reentry of the vaporized droplets) results in expulsion of a second plume, which is primarily responsible for ablation efficiency. During method development, the data obtained from the first well positions of a 96-well plate varied greatly compared to other columns. A possible cause for this phenomenon is air currents created by movement of the LAESI stage, which might disrupt the ion sampling. To offset this observed stage effect, we left the initial wells in each column either blank or filled with water. The ESI mass spectrograph was obtained using a Thermo Fisher LTQ XL mass spectrometer, containing an atmospheric pressure ionization stack with a tube lens and skimmer, three multipoles, a single linear trap configuration, and a set of 2 electron multipliers with conversion dynodes. The mass spectrometer was connected to a Protea LAESI DP-1000 instrument with an ESI electrospray emitter for ambient ionization. The collected data points were exported to Gubbs Mass Spec Utilities (60) and processed using the Generic chromatographic viewer for individual *m/z* ratios (Thermo Fisher Scientific).

### LC-HRESIMS.

For final confirmation of our findings from LAESI-MS, we acquired HRESIMS spectra using an Agilent 6520 quadrupole time of flight (qTOF) mass spectrometer equipped with an Agilent 1290 HPLC system at the Life Sciences Institute core facility. For this analysis, cultures were grown in IDM for 5 to 6 h, and 20 ml of culture medium was collected by filter sterilization through a 0.22-µm-pore filter and then stored at −80°C. Culture media were dried down (Labconco freeze dry system), and the pellet was resuspended in 1 ml of ACS-grade methanol. Undissolved particulates were removed by centrifugation at 5,000 × *g* for 1 min, and the supernatant was used for analysis. Reverse-phase HPLC was performed using Luna 5-µm C_18(2)_ 100-Å, 100- by 2-mm HPLC column and a solvent gradient from 30% water (plus 0.1% formic acid) to 90% methanol (plus 0.1% formic acid) over 16 min.

### Murine infections.

Five 6- to 8-week-old, female DBA/J2 mice (Jackson Laboratories) were infected intratracheally with 1.5 × 10^5^ spores as described by Heffernan et al. (49). Prior to infection, spores were passed through a 3.1-µm-pore glass microfiber filter (National Scientific Company) to increase purity and reduce clumping and then counted by hemocytometer. Mice were monitored for morbidity for 14 days postinfection and euthanized when moribund. All mouse experiments were performed using protocols (PRO00007362) approved by the University of Michigan Committee on the Use and Care of Animals.
