# Erratum for Hagan et al., “Petrobactin Is Exported from *Bacillus anthracis* by the RND-Type Exporter ApeX”

**DOI:** 10.1128/mBio.01686-17

**Published:** 2017-10-17

**Authors:** A. K. Hagan, A. Tripathi, D. Berger, D. H. Sherman, P. C. Hanna

**Affiliations:** a Department of Microbiology and Immunology, University of Michigan, Ann Arbor, Michigan, USA; b Life Sciences Institute, Department of Medicinal Chemistry, University of Michigan, Ann Arbor, Michigan, USA; c Life Sciences Institute, Department of Medicinal Chemistry, Department of Chemistry, Department of Microbiology and Immunology, University of Michigan, Ann Arbor, Michigan, USA

## ERRATUM

Volume 8, no. 5, e01238-17, 2017, https://doi.org/10.1128/mBio.01238-17. Some of the petrobactin component structures in Table 3 were corrupted during file conversion. We apologize for not detecting and correcting this error before publication. The corrected Table 3 is below.

**TABLE 3:**
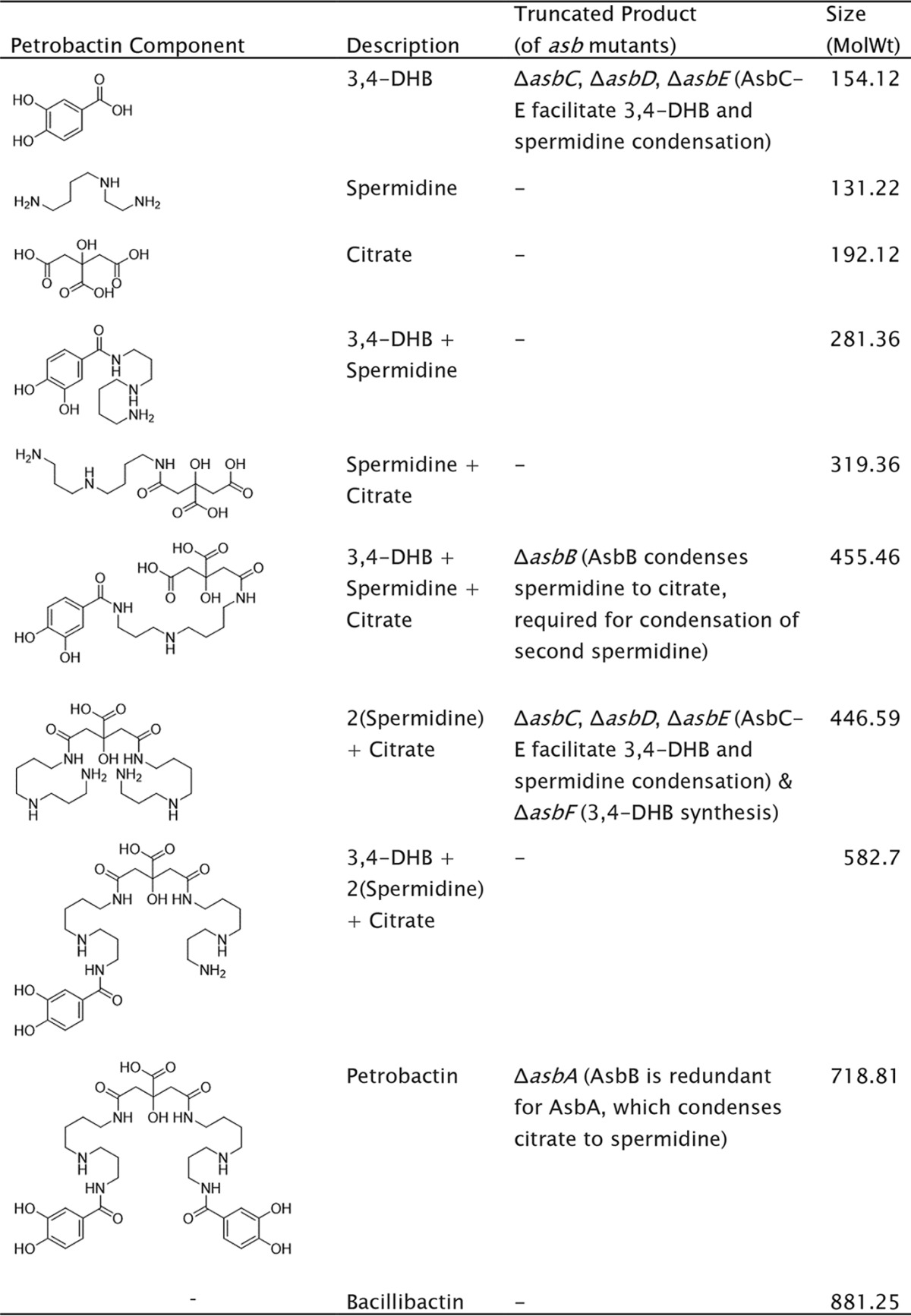
Structures and molecular weights of predicted petrobactin components present in individual *asbA* to -*F* knockout mutants^a^

a Nusca et al. (43).

