# Supplementary figures and images for: Petrobactin Is Exported from Bacillus anthracis by the RND-Type Exporter ApeX
Source: mBio. 2017 Sep 12;8(5):e01238-17. doi: 10.1128/mBio.01238-17 (PMC5596346; doi:10.1128/mBio.01238-17)

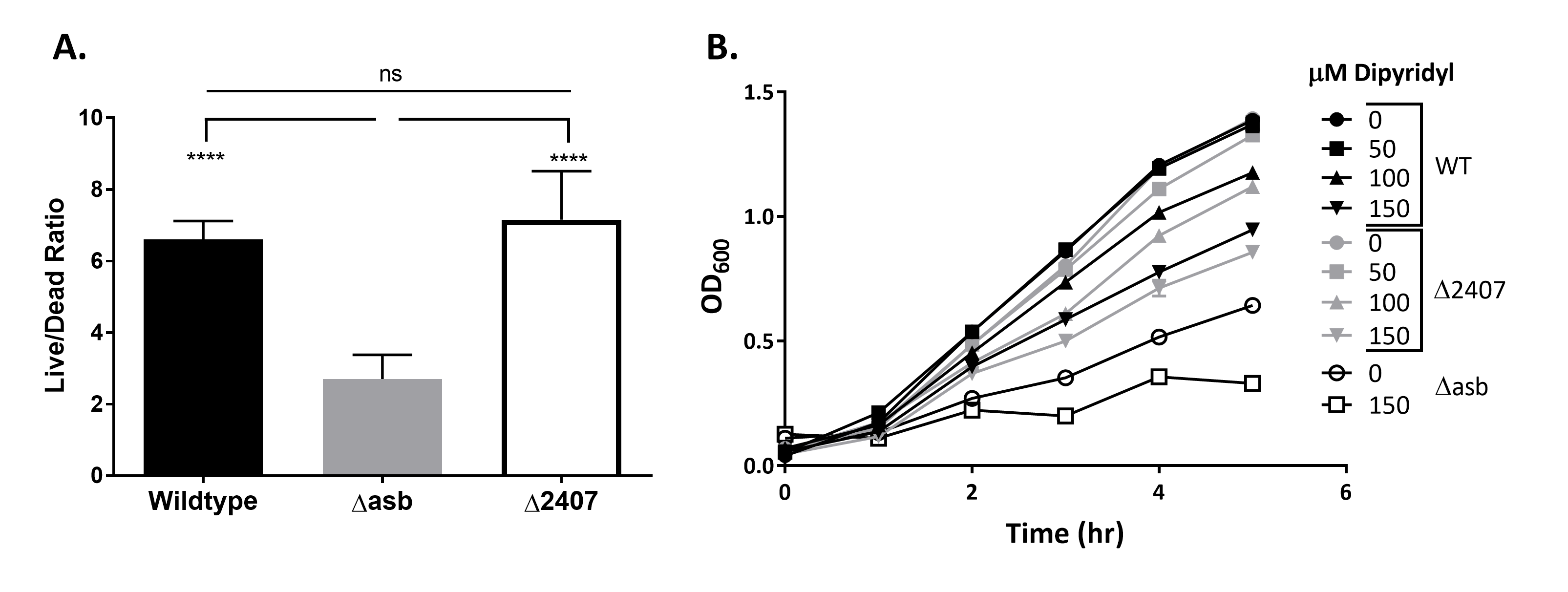

Supplement: FIG S1 [file mbo004173478sf1.tif]

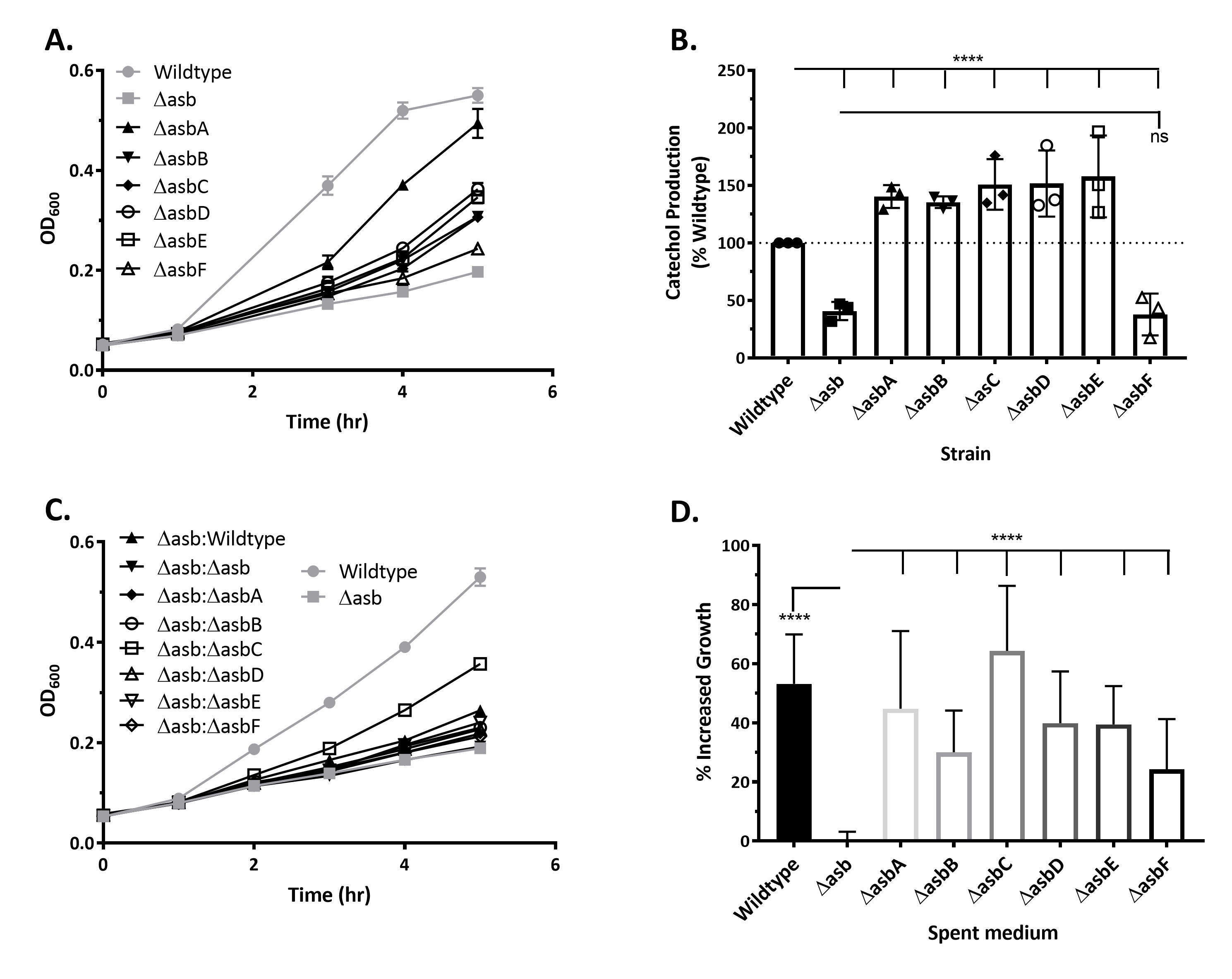

Supplement: FIG S2 [file mbo004173478sf2.tif]

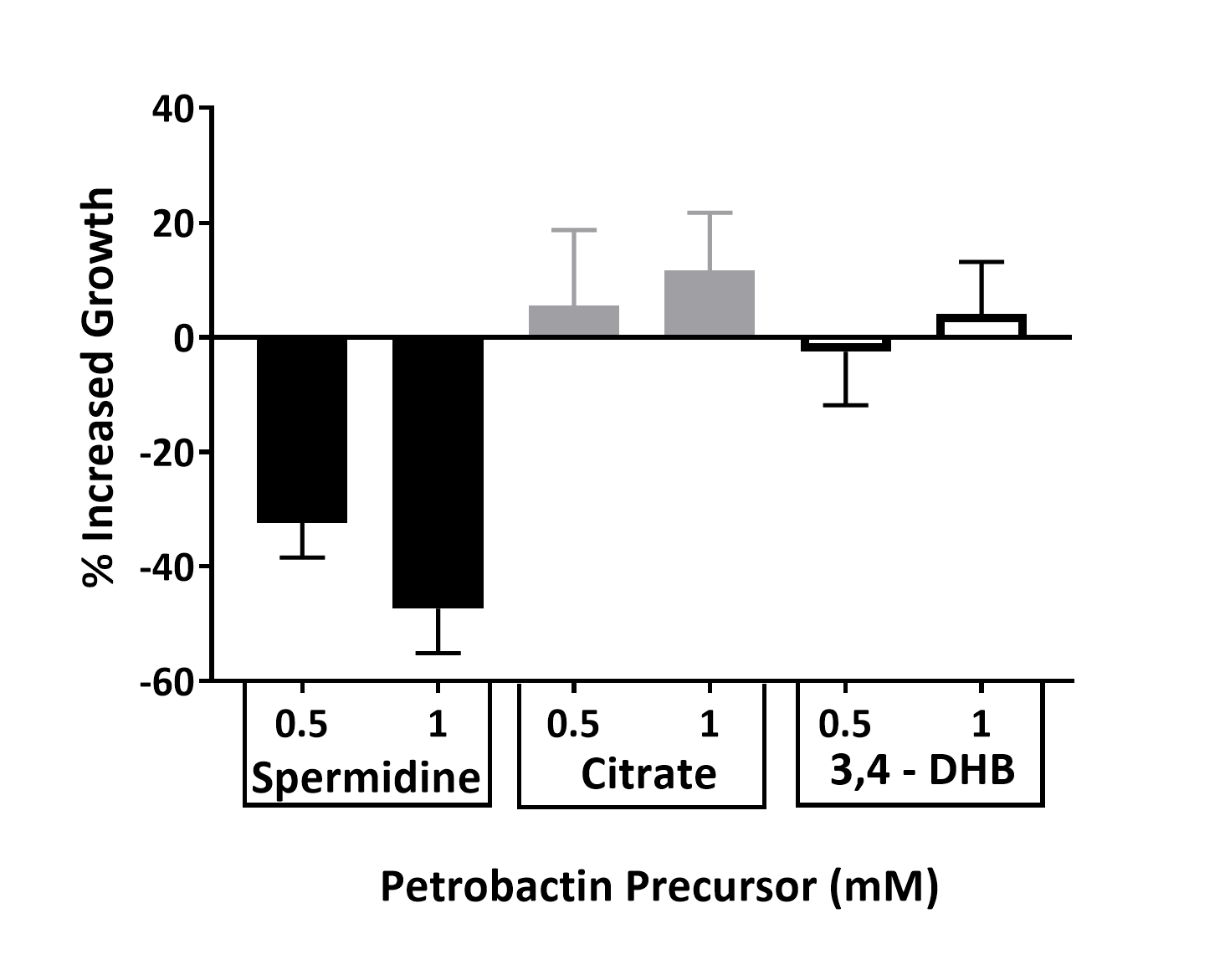

Supplement: FIG S3 [file mbo004173478sf3.tif]

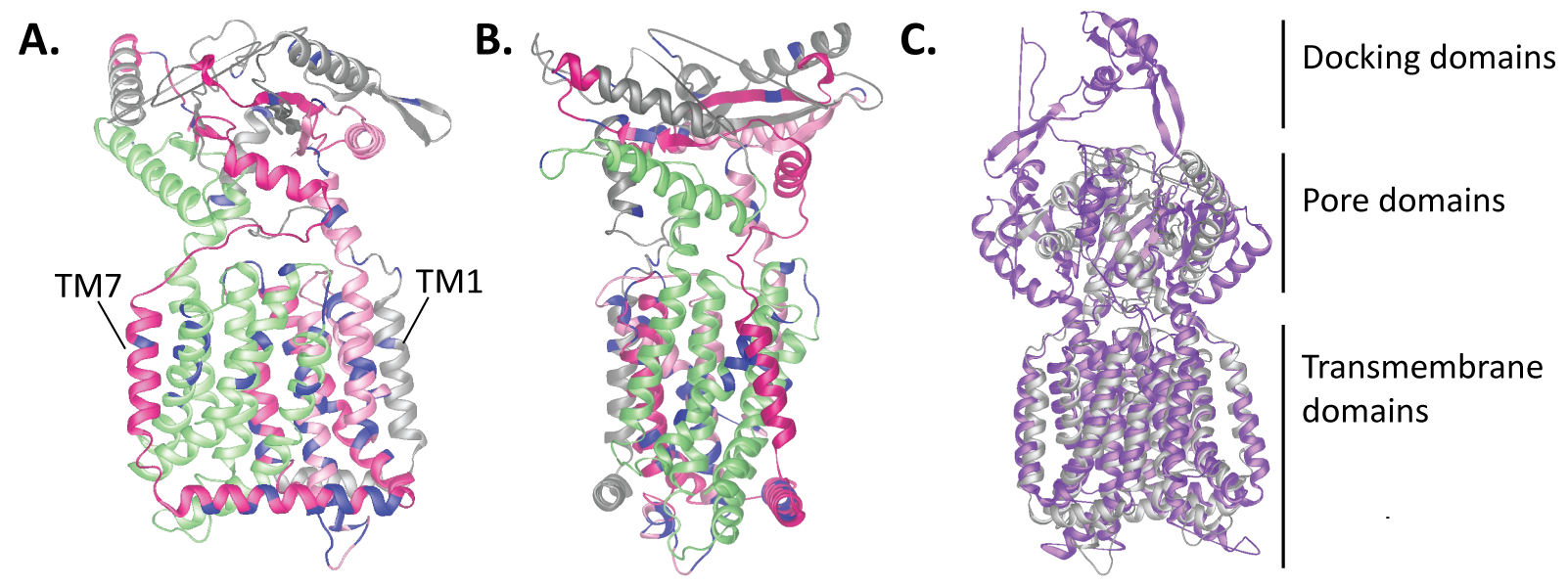

Supplement: FIG S4 [file mbo004173478sf4.tif]
