# Supplementary material for: Petrobactin Is Exported from Bacillus anthracis by the RND-Type Exporter ApeX
Source: mBio. 2017 Sep 12;8(5):e01238-17. doi: 10.1128/mBio.01238-17 (PMC5596346; doi:10.1128/mBio.01238-17)
Supplement: TABLE S1 [file mbo004173478st1.docx]

Supplementary Table 1. Strains of *B. anthracis* Sterne 34F2 used in this work.

| **Strain** | **Relevant characteristics** | **Reference** |
| --- | --- | --- |
| *Bacillus anthracis* Sterne 34F2 | Wild type (pXO1+, pXO2-) | Sterne, 1939 |
| 34F2, ΔasbABCDEF | Petrobactin biosynthesis mutant | Lee et al., 2007 |
| 34F2, ΔasbA |  | Pfleger et al., 2008 |
| 34F2, ΔasbB |  | Pfleger et al., 2008 |
| 34F2, ΔasbC |  | Pfleger et al., 2008 |
| 34F2, ΔasbD |  | Pfleger et al., 2008 |
| 34F2, ΔasbE |  | Pfleger et al., 2008 |
| 34F2, ΔasbF |  | Pfleger et al., 2008 |
| 34F2, ΔfpuA | Petrobactin receptor mutant | Carlson, et al., 2010 |
| 34F2, ΔGBAA_3296 |  | This work |
| 34F2, ΔGBAA_1642 |  | This work |
| 34F2, Δ1642Δ3296 |  | This work |
| 34F2, ΔGBAA_2407 |  | This work |
| 34F2, ΔGBAA_1302 |  | This work |
| 34F2, Δ2407Δ1302 |  | This work |
| 34F2, Δ2407 pAH001 | ΔGBAA2407 + pAH001 | This work |
| 34F2, Δ2407 p2407 | ΔGBAA2407 + 2407pAH001 | This work |
| 34F2, ΔGBAA_0181 |  | This work |
| 34F2, ΔGBAA_0787 |  | This work |
| 34F2, ΔGBAA_4961 |  | This work |
| 34F2, ΔGBAA_5668 |  | This work |
| 34F2, ΔGBAA_0835 |  | This work |
| 34F2, ΔGBAA_1858 |  | This work |
| 34F2, ΔGBAA_2004 |  | This work |
| 34F2, ΔGBAA_3157 |  | This work |
| 34F2, Δ0835Δ1858 |  | This work |
| 34F2, Δ4961Δ5668 |  | This work |
| 34F2, ΔGBAA_0852 |  | This work |
| 34F2, ΔGBAA_0528 |  | This work |
| 34F2, ΔGBAA_1652 |  | This work |
| 34F2, ΔGBAA_5411 |  | This work |
| 34F2, ΔGBAA_2346 |  | This work |
| 34F2, ΔGBAA_4504 |  | This work |
| 34F2, ΔGBAA_4595-96 |  | This work |
| 34F2, Δ0528Δ0852Δ5411 |  | This work |
