# Supplementary material for: Petrobactin Is Exported from Bacillus anthracis by the RND-Type Exporter ApeX
Source: mBio. 2017 Sep 12;8(5):e01238-17. doi: 10.1128/mBio.01238-17 (PMC5596346; doi:10.1128/mBio.01238-17)
Supplement: TABLE S2 [file mbo004173478st2.docx]

| **Supplementary Table 2. Primers used to generate mutant strains used in this work.** | |
| --- | --- |
| **Primer** | **Sequence (5'→3')** |
| GBAA_1302-GibsP1 | AAGTTGATAAGTGGGGGAAGGTAACTGGTGCGTTTGCTTTTACTG |
| GBAA_1302-GibsP2 | AAAGCAAACGCACCAGTTACCTTCCCCCACTTATCAACTTTATGTACA |
| GBAA_1302-GibsP3 | GAACAAAAGCTGGAGCTCCACCGCGGTGGCCGGTGCTCGTTATAAGTTTACTTGTTTTTTG |
| GBAA_1302-GibsP4 | GATATCAGATCTGACGTCTCTAGAGCGGCCCCCGATACTTCTTGTATTTTGTAATACTCATC |
| GBAA_1302-P5 | GGCGTTACAGTGATGG |
| GBAA_1302-P6 | GTCATTTCGCACCATTC |
| GBAA_2407-P1 | CATCCGTTACATATGTTAGGGGAAGTACAAAAGGTGGATGC |
| GBAA_2407-P2 | GCATCCACCTTTTGTACTTCCCCTAACATATGTAACGGATG |
| GBAA_2407-GibsP3 | GAACAAAAGCTGGAGCTCCACCGCGGTGGCCTTTAATTGTTCTTCAGATAAAGATAGTTTTGC |
| GBAA_2407-GibsP4 | GATATCAGATCTGACGTCTCTAGAGCGGCCGCGTAATATCAACATTTACTGTCATACTAAACG |
| GBAA_2407-P5 | CCAATAAAAAGAAACGATTTC |
| GBAA_2407-P6 | CTCGGGATCATTTCCTC |
| 2407 MJcomp F | CAATAAATGTCCTGTTAAAGGAGGTGTTTTCTAGAATGAAAAAGCATCCGTTACATATG |
| 2407 MJcomp R | GAAGCTCGGCGGATTTGTCCTACTCAAGCTCTATGCATCCACCTTTTGTACTTCTTC |
| GBAA-1642 P1 | ATAGTAATGTAATGAAGAAAATCCCCTTGAGCAAGTA |
| GBAA-1642 P2 | TCTTCATTACATTACTATTTTGCTCCTTTTTTCAACG |
| GBAA-1642 P3 | CGCGCGGCCGCTACTACATTCATTCGTTGCAAAAAA |
| GBAA-1642 P4 | CGCGCGGCCGCGTTATTACATGTTTGCATTTAATCC |
| GBAA_1642-GibsP1 | AAAAGTTTATTTATCCCATTAAACATGGCAGGAGAGAAG |
| GBAA_1642-GibsP2 | GCCATGTTTAATGGGATAAATAAACTTTTAATTGGTTTTTCTCTTAATT |
| GBAA_1642-GibsP3 | GGAACAAAAGCTGGAGCTCCACCGCGGTGGCCTTATTTTCTGGAGATATCGGTGTAACTG |
| GBAA_1642-GibsP4 | GGATATCAGATCTGACGTCTCTAGAGCGGCCCTATTCAACAAACTAAATCGTTTTATTCTCG |
| GBAA_1642 P5 | GAGGCAAATGGATGATAG |
| GBAA_1642 P6 | CATTTAATCCTTACAAAATTAAGC |
| GBAA_3296 P1 | GAAATCAAATATGTAGAATAGAAGATGAAAAATGATAT |
| GBAA_3296 P2 | CATCTTCTATTCTACATATTTGATTTCCCCTTTTATTTG |
| GBAA_3296 P3 | CGCGCGGCCGCAGCAGTTAGCAAAAGCTAACTACTC |
| GBAA_3296 P4 | CGCGCGGCCGCTTTTCAAGGAAAAGGTATAGGTAAA |
| GBAA_3296 P5 | CATCATTTTGGAACATACTTAATAA |

| GBAA_3296 P6 | AATTATTGGATTTATAGACTTATGA |
| --- | --- |
| GBAA_0181-GibsP1 | CGGGTGCATCTCTAGCACAGGAGGTTTAATGTATAAAGAAGAGCTGATTCA |
| GBAA_0181-GibsP2 | CTTCTTTATACATTAAACCTCCTGTGCTAGAGATGCACCCGTTT |
| GBAA_0181-GibsP3 | GAACAAAAGCTGGAGCTCCACCGCGGTGGCGACCAACTTCAAGTAGTAAAGAGGCAC |
| GBAA_0181-GibsP4 | GATATCAGATCTGACGTCTCTAGAGCGGCCGATCAATATCTTCTAACATGCTCGTCG |
| GBAA_0181 P5 | GCCCATACTGTTATCTATGC |
| GBAA_0181 P6 | CGTTTATAGTATGTCGATGCTG |
| GBAA_0787-GibsP1 | GCAATAAGTAGTAGAGAGGGGCCAATGTAACAGTCAAGGAAGG |
| GBAA_0787-GibsP2 | CCTTGACTGTTACATTGGCCCCTCTCTACTACTTATTGCTTTCCTGTTC |
| GBAA_0787-GibsP3 | GAACAAAAGCTGGAGCTCCACCGCGGTGGCCGATCTAGAATATCGCGAAGACGATC |
| GBAA_0787-GibsP4 | GATATCAGATCTGACGTCTCTAGAGCGGCCGGAAAAACGATAAATCGTTATATGAAATACC |
| GBAA_0787 P5 | CGTACCGATGTTCGC |
| GBAA_0787 P6 | CAATTTTTAAGCCATACGG |
| GBAA_4961 P1 | GCGATTCTGTAATTTTATCGCACGTTATTTATATGAGCACTTTC |
| GBAA_4961 P2 | GAAAGTGCTCATATAAATAACGTGCGATAAAATTACAGAATCGC |
| GBAA_4961 P3 | GCGGCCGCGCGCCAAGCTGGACAAGTTTCTC |
| GBAA_4961 P4 | GCGGCCGCGCGCGAAGGCTAAGTGTCCTCATC |
| GBAA_4961 P5 | GAACAACTATTCCAGCGC |
| GBAA_4961 P6 | CGGCTGGAAACATCC |
| GBAA_4504-GibsP1 | GTAACTCTTTATATGCAGGGGGTGCGATTTTATGGAAGAAGAAAAAAAC |
| GBAA_4504-GibsP2 | CTTCTTCCATAAAATCGCACCCCCTGCATATAAAGAGTTACGTAAAAAGTC |
| GBAA_4504-GibsP3 | GAACAAAAGCTGGAGCTCCACCGCGGTGGCCCGGCAACTGTGTTTGAAGTTG |
| GBAA_4504-GibsP4 | GATATCAGATCTGACGTCTCTAGAGCGGCCGTTGCCCACAATATTTGTGAGCAAC |
| GBAA_4504 P5 | CGGCAACTGTGTTTG |
| GBAA_4504 P6 | GTTGCCCACAATATTTGTG |
| GBAA_2346 GibsP1 | GACTTTAGGCTAAAACATGCGTCTTTTTATCATTTGCATATGGAGGC |
| GBAA_2346 GibsP2 | CATATGCAAATGATAAAAAGACGCATGTTTTAGCCTAAAGTCCTATC |
| GBAA_2346 GibsP3 | GAACAAAAGCTGGAGCTCCACCGCGGTGGCGTAATGCAGGAGAGGCCCCTG |
| GBAA_2346 GibsP4 | GATATCAGATCTGACGTCTCTAGAGCGGCCCTTTTTTGCCTAATTGCTGTTTTACAAAAAAC |
| GBAA_2346 P5 | CACACAACCGGAGACTG |
| GBAA_2346 P6 | GCCTTTACAATATTGTGAAACTAC |

| GBAA_0835 P1 | GAACAAAAGATGTAATAAAACCCCTTACAACGGTAAG |
| --- | --- |
| GBAA_0835 P2 | GGGGTTTTATTACATCTTTTGTTCTTTTATCGGTTTC |
| GBAA_0835 P3 | CGCGCGGCCGCATAGAGGAATATCGTGAAATACAAG |
| GBAA_0835 P4 | CGCGCGGCCGCCCCTTTGTATTTCATATTGCTATTG |
| GBAA_0835 P5 | ACGCAAGTAATAGATGCGGTATTTC |
| GBAA_0835 P6 | TTACCCCCATGAAAATCACCTCCCC |
| GBAA_1858 P1 | GTGAAAAATGTAACTAAGTGAATGAAAAAAGGATG |
| GBAA_1858 P2 | CACTTAGTTACATTTTTCACACCTTCTTTCTTTAG |
| GBAA_1858 P3 | CGCGCGGCCGCTGAGAAATTAGTAAGTTTTGTTAGT |
| GBAA_1858 P4 | CGCGCGGCCGCTCCATCATAATATGACGAGAGGTTT |
| GBAA_1858 P5 | TCATGTTAGAAAGGCTAACTCTTCT |
| GBAA_1858 P6 | GTTAGAAACAAAGTTTAAAAACAAA |
| GBAA_2004 P1 | GATTTAAAATGTAAAGTCTTTACAAATAAAAAAGAG |
| GBAA_2004 P2 | GTAAAGACTTTACATTTTAAATCTTCTCCTTTTATAT |
| GBAA_2004 P3 | CGCGCGGCCGCCTTTTGGGATATTAGGTGGTAAATA |
| GBAA_2004 P4 | CGCGCGGCCGCCAAATTATTGCGACGTTAGTCTATA |
| GBAA_2004 P5 | GTATTTCATTTATCCCTTACGGGCC |
| GBAA_2004 P6 | ATCGGGAAAGCTGATGAGAAGATTC |
| GBAA_3157-GibsP1 | CAAAATAAAAAAAGACTACTTATGCAGTACTTTATCTTTAAATTTCTAAAGATTTTTTAAAG |
| GBAA_3157-GibsP2 | GAAATTTAAAGATAAAGTACTGCATAAGTAGTCTTTTTTTATTTTGAACGAATACTATTTC |
| GBAA_3157-GibsP3 | GAACAAAAGCTGGAGCTCCACCGCGGTGGCCTCTTTTAGAACTTCATATAAAGGATTTGAAATC |
| GBAA_3157-GibsP4 | GATATCAGATCTGACGTCTCTAGAGCGGCCGACATTTTTATCCAGCTTTAGAACCTTTTATG |
| GBAA_3157 P5 | CTCTTTTAGAACTTCATATAAAGG |
| GBAA_3157 P6 | GACATTTTTATCCAGCTTTAG |
| GBAA_0852-GibsP1 | GTATTACTAAAGTTAAAGTGGCGAGCTGTTATTCAAAATCCAGAAATTTTAAT |
| GBAA_0852-GibsP2 | GGATTTTGAATAACAGCTCGCCACTTTAACTTTAGTAATACTGAAAACAATGTAATC |
| GBAA_0852-GibsP3 | GAACAAAAGCTGGAGCTCCACCGCGGTGGCCGATTTCTTATGAAGTTTTTATTTTGTATGC |
| GBAA_0852-GibsP4 | GATATCAGATCTGACGTCTCTAGAGCGGCCCATCACGCGTACTTCTTTTCCTTTTTTC |
| GBAA_0852 P5 | GATTTCTTATGAAGTTTTTATTTTG |
| GBAA_0852 P6 | CAAAGTAATATTGAAAACCGAC |
| GBAA_0528-GibsP1 | GTTAAACCATATCGATGGCATATAACAGAAACAGCTCCTTTAGCG |

| GBAA_0528-GibsP2 | CTGTTTCTGTTATATGCCATCGATATGGTTTAACAAATTGC |
| --- | --- |
| GBAA_0528-GibsP3 | GAACAAAAGCTGGAGCTCCACCGCGGTGGCAGTCTTGTAATCGGAGCGAATGATC |
| GBAA_0528-GibsP4 | GATATCAGATCTGACGTCTCTAGAGCGGCCAAGTACAGTTCGCAAAAATTCGTAAGC |
| GBAA_0528 P5 | CAATTTTAAAAGGGACACAG |
| GBAA_0528 P6 | GAAGAAAGAAGTTACACGAAAA |
| GBAA_1652-GibsP1 | CTTCTTAAAACAAATAAAAACGGGAAACTTAGTGAAGAATCTACCATTACTTAATAG |
| GBAA_1652-GibsP2 | GATTCTTCACTAAGTTTCCCGTTTTTATTTGTTTTAAGAAGTGCGGAATAAG |
| GBAA_1652-GibsP3 | GAACAAAAGCTGGAGCTCCACCGCGGTGGCGCATTTTTACTTAGGAGGATTACAATGATTAAAG |
| GBAA_1652-GibsP4 | GATATCAGATCTGACGTCTCTAGAGCGGCCCTTGTGTAACCTCCCATGTAAGCTGTTGTAAT |
| GBAA_1652 P5 | GCATTTTTACTTAGGAGGATTAC |
| GBAA_1652 P6 | CTTGTGTAACCTCCCATG |
| GBAA_5411-GibsP1 | CTTACTATAAACCGGGATACAGCAGGTTATACGAAGCG |
| GBAA_5411-GibsP2 | GTATAACCTGCTGTATCCCGGTTTATAGTAAGAAAAAAATTTACGTAGC |
| GBAA_5411-GibsP3 | GAACAAAAGCTGGAGCTCCACCGCGGTGGCGAATTTTATGATGCGCTTCAAACG |
| GBAA_5411-GibsP4 | GATATCAGATCTGACGTCTCTAGAGCGGCCCCTATATATCGACGGGACCTTCCG |
| GBAA_5411 P5 | CCAAGAAGCGCTGAG |
| GBAA_5411 P6 | GTAAAGAAGCACTCTAATGTCTTC |
| GBAA_4595/96-GibsP1 | GTGAAGTAGTAAGGGAAGAGGAACAGAAGAACGTGTTACCACTGAGTTAG |
| GBAA_4595/96-GibsP2 | GGTAACACGTTCTTCTGTTCCTCTTCCCTTACTACTTCACTACTTTCCATAC |
| GBAA_4595/96-GibsP3 | GAACAAAAGCTGGAGCTCCACCGCGGTGGCGAGCTATATGGAACGATAATGAAGAAAGAAG |
| GBAA_4595/96-GibsP4 | GATATCAGATCTGACGTCTCTAGAGCGGCCCTAGCATAAAGAAAACTACAATTGAGGGG |
| GBAA_4595/96 P5 | GAGCTATATGGAACGATAATG |
| GBAA_4595/96 P6 | CTAGCATAAAGAAAACTACAATTG |
| GBAA_5668-GibsP1 | GGTGAAAGAAATTTCGAAGGAAACGAAACAACGAGAATTAGTATAAGATG |
| GBAA_5668-GibsP2 | CTAATTCTCGTTGTTTCGTTTCCTTCGAAATTTCTTTCACCTTGCC |
| GBAA_5668-GibsP3 | GAACAAAAGCTGGAGCTCCACCGCGGTGGCCGAGACAAGAGAACTTCATCCAAACG |
| GBAA_5668-GibsP4 | GATATCAGATCTGACGTCTCTAGAGCGGCCCTTGAAAACGAAATGCCGTAATGG |
| GBAA_5668 P5 | CGAGACAAGAGAACTTCATC |
| GBAA_5668 P6 | GCTTGAAAACGAAATGC |
